# Epidemiological Characteristics of Work-Related Ocular Trauma in Southwest Region of China

**DOI:** 10.3390/ijerph120809864

**Published:** 2015-08-19

**Authors:** Mingming Cai, Jie Zhang

**Affiliations:** 1 Department of Ophthalmology, The Ninth People’s Hospital of Chongqing, 69 Jia Ling Road, Chongqing 400700, China; E-Mail: mm198222@126.com; 2 Department of Urology, The Ninth People’s Hospital of Chongqing, 69 Jia Ling Road, Chongqing 400700, China

**Keywords:** occupational health, eye injuries, work-related, ocular trauma, epidemiology

## Abstract

*Purpose*: To determine the epidemiological characteristics of work-related eye injury in representative southwest region of China. *Methods*: Patients with eye injuries treated at the Ninth People’s Hospital of Chongqing from 1 January 2014 to 31 December 2014 were included in the current study. All patients completed a comprehensive examination and interview. Demographic characteristics and injury details were recorded. The International Classification of Diseases (ICD-10) and Birmingham Eye Trauma Terminology (BETT) were used. *Results*: The average age of eye injury patients was 37.52 years and the majority were male. Among the 1055 total patients, approximately 42.9% of the injuries were work-related. The highest proportion of occupational eye trauma was observed in the group between 36 and 45 years of age. Occupational ocular trauma occurred more frequently in summer, with most from 16:00 to 18:00. Metal was the most common injury cause. Foreign body on external eye was the most common diagnosis. Workers in the manufacturing industry without pre-work safety training or eye protection were far more likely to suffer from occupational ocular trauma than those with training and protection. *Conclusions*: This study provides insight into the epidemiological characteristics of occupational ocular trauma in southwest region of China. The current findings might be considered as a baseline for future research on regional work-related eye injuries. Our findings will provide valuable information for further development of preventive strategies.

## 1. Introduction

Occupational ocular trauma is an important cause of preventable vision loss worldwide, with significant socioeconomic impact. It is a major cause of emergency ophthalmic visits and accounts for a substantial proportion of eye injuries, many of which occur in the workplace [1,2].

More than 65,000 work-related eye injuries and illness are reported in the United States annually [3]. More than 2000 eye injuries occur at work daily and approximately 10%–20% of eye injuries result in temporary or permanent vision loss [4]. In Hong Kong, the annual incidence of occupational eye injuries was estimated to be around 8000 cases, or around 125 cases per 100,000 population, and they account for 8% of all occupational injuries recorded in selected hospitals [5]. Since the incidence of ocular trauma varies by population, it is necessary to conduct epidemiological studies in each region [6]. 

Although research on occupational ocular trauma has been done in some areas, it has not been well studied in other industrialized countries, like China, where about one-sixth of the people of the world live. China is the largest developing country in the world. Occupational ocular trauma is a major problem in its development [7]. Chongqing Municipality is considered an economic and industrial hub of southwest region of China. We selected Beibei District of Chongqing as the study site to conduct an epidemiological study of work-related eye injuries in Southwest China. The population and occupational structure of this district is relatively stable, and the socioeconomic status in the southwest region of China is intermediate. We studied the epidemiological characteristics of work-related ocular trauma presenting to the Ninth People’s Hospital of Chongqing. This is the first report on epidemiology of occupational eye trauma in the Southwest region of China. Our findings will provide valuable information for the further developing effective prevention strategies in Southwest China and other developing countries.

## 2. Methods

Beibei District is one of the 40 administrative districts in Chongqing Municipality. One of the characteristics of the district is the industrial base, which includes machinery, household electrical appliance, biomedical device, fibreglass and optical instrument manufacture. The Ninth People’s Hospital of Chongqing is a hospital with comprehensive ophthalmology services. It is the major eye trauma centre of Beibei District. Almost all patients requiring specialist ocular treatment are referred to the ophthalmology department in this area. Study subjects included both out-patients and patients hospitalized in this hospital. Patients who were initially treated by other services, but with concomitant eye injuries were also sent to the Ophthalmology Department for consultation and necessary treatment. Therefore, these data are considered to represent the major occupational ocular injuries in this area.

Eye injuries treated at the Ninth People’s Hospital of Chongqing from 1 January 2014 to 31 December 2014 were included in the current study. The study was approved by the Institutional Ethics Committee of the Ninth People’s Hospital of Chongqing and followed the tenets of the Declaration of Helsinki. Patients <15 years old or re-admitted for treatment of previous eye injuries were excluded. Study subjects included workers from both rural and urban areas. During the one year study period, the population and industrial structure in this area were stable.

All patients who presented to the Ophthalmology Department received a comprehensive ocular examination and a face-to-face interview by trained medical workers, using a structured questionnaire. The following information were collected: patients’ demographic characteristics, media of injuries, safety provisions, injury details, clinical findings, treatment received. To minimize information bias, interviewers were trained on delivering the interviews in a standardized manner. For the purpose of classification, the International Classification of Diseases (ICD-10) [8] and Birmingham Eye Trauma Terminology System (BETTS) [9,10] were used. The data were analyzed using SPSS version 17.0 software (SPSS Inc., Illinois, IL, USA). Statistical analysis of quantitative data was performed for all variables. Chi-square tests were used to compare proportions. Confidence intervals of 95% were employed. All *p*-values were two-sided and P-values less than 0.05 were considered statistically significant.

## 3. Results

**Figure 1:**
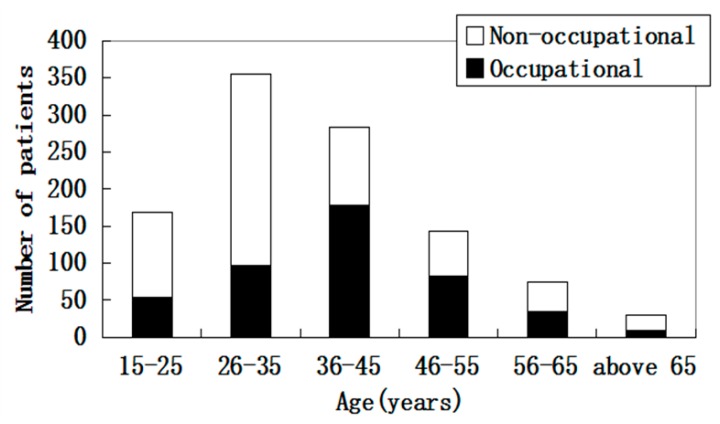
Age distribution of occupational and non-occupational eye trauma.

From 1 January 2014 to 31 December 1197 eye injuries from 1055 patients were treated at the Ophthalmology Department of the Ninth People’s Hospital of Chongqing. Demographically (Table 1) all patients were 15 years of age or older. The average ages were 39.82 ± 11.16 and 35.79 ± 12.69 for the occupational and non-occupational group. Of all the cases of ocular trauma, 26-35 years was the most common age group, followed by the 36–45 years and 15–25 years age groups. However, occupational ocular trauma occurred more frequently in people between 26 and 55 years of age, the highest proportion was observed in the group between 36 and 45 years of age. The age distribution shifted toward older ages in the work-related injury group (Figure 1). 

**Table 1:** Demographic characteristics of occupational and non-occupational eye trauma.

Variable	Total, n(%) 1055 (100.0)	Occupational, n (%) 453 (42.9)	Non-Occupational, n (%) 602 (57.1)	*p* Value
Gender				
Male	848 (80.3)	417 (92.1)	431 (71.6)	0.000
Female	207 (19.7)	36 (7.9)	171 (28.4)	
Age (yr), mean ± SD	37.52 ± 12.21	39.82 ± 11.16	35.79 ± 12.69	0.000
Education level				
No school or primary school	247(23.4)	156 (34.4)	91 (15.1)	0.000
Middle school	385(36.5)	219 (48.4)	166 (27.6)	
High school	303(28.7)	67 (14.8)	236 (39.2)	
College or above	120(11.4)	11 (2.4)	109 (18.1)	

Data are presented as mean ± SD or percentages.

Male patients constituted the majority of eye injury patients. The proportion of males in the occupational group was significantly greater than in the non-occupational group. The educational level was lower in the occupational group (*p* < 0.05). As shown in Table 2, most injured workers were in manufacturing (60.7%), followed by construction (19.6%), agriculture (8.2%), and services (4.0%). Machine guarding is not widely carried out. Workers in the manufacturing industry without pre-work safety training or eye protection were far more likely to suffer from occupational ocular trauma than those with training and protection.

**Table 2:** Characteristics of occupational eye trauma.

Characteristics	n (%)
*Employment*	
Permanent	155 (34.2)
Temporary	298 (65.8)
*Safety training*	
Yes	102 (22.5)
No	351 (77.5)
*Working with eye protection*	
Yes	32 (7.1)
No	421 (92.9)
*Machine guarding*	
Yes	114 (25.2)
No	339 (74.8)
*Industrial sector*	
Manufacturing	275 (60.7)
Construction	89 (19.6)
Agriculture	37 (8.2)
Services	18 (4.0)
Other	34 (7.5)

Data are presented as percentages

**Figure 2:**
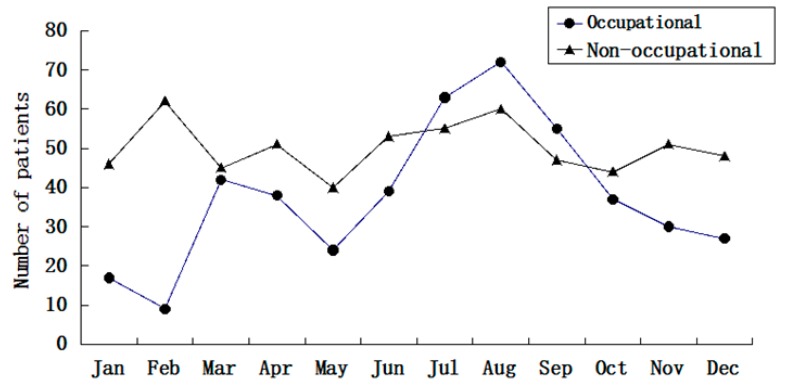
Seasonal distribution of occupational and non-occupational eye trauma.

**Figure 3:**
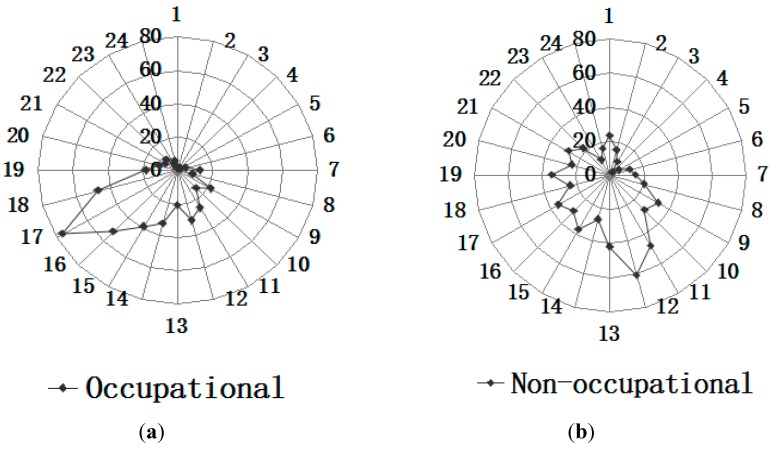
(**a**,**b**) Incidence times of occupational and non-occupational eye trauma over a one-day period.

The seasonal distribution of occupational ocular trauma, with a maximum occurring during the summer months, and a minimum occurring during the winter months, is seen in Figure 2. There was a small peak in March and April. However, there was no obvious peak for the seasonal distribution of non-occupational ocular trauma. The eye injuries occurred both day and night, with most work-related injuries occurring from 16:00 to 18:00 (165 cases, 36.4%) and most non-occupational eye injuries occurring from 11:00 to 13:00 (151 cases, 25.1%) (Figure 3). 

**Figure 4:**
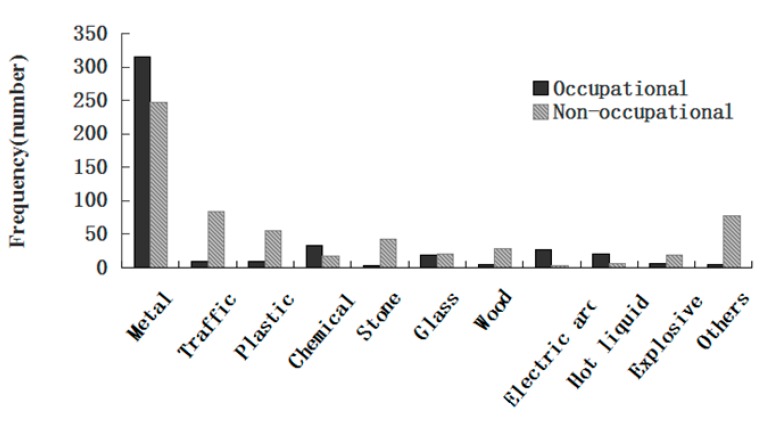
Causes of occupational and non-occupational eye trauma.

Figure 4 shows the causes of eye injuries in both groups. The most common causes of ocular injuries were metal (562 patients, 53.3% of total ocular injuries), traffic (93 patients, 8.8% of total ocular injuries) and plastic (66 patients, 6.2% of total ocular injuries). Metal was the most common cause for the occupational injury group, with 315 (69.5%) cases, followed by 34 (7.5%) cases of chemical, 27 (6.0%) cases of electric arc, 21 (4.6%) cases of hot liquid, 19 (4.2%) cases of glass, 10 (2.2%) cases of plastic, 9 (2.0%) cases of traffic and 6 (1.3%) cases of explosive exposure. For the non-occupational injury group, the most common medium was also metal (247 patients, 41%), but followed by traffic with 84 (14%) cases, plastic with 56 (9.3%) cases, stone with 43 (7.1%) cases, wood with 28 (4.7%) cases, glass with 20 (3.3%) cases, explosive with 19 (3.2%) cases, and chemical with 18 (3.0%) cases.

As shown in Table 3, the most common diagnoses of all the eye injuries in our study were foreign body on external eye (397, 37.6%), contusion of eye and adnexa (226, 21.4%), superficial wound of eye and adnexa (193, 18.3%), open wounds of ocular adnexa (115, 10.9%), burn confined to eye and adnexa (79, 7.5%), open wounds of globe (27, 2.6%)and others (18, 1.7%). 

**Table 3:** Diagnosis of occupational and non-occupational eye trauma.

Diagnosis	Total, n (%)	Occupational, n (%)	Non-occupational, n (%)	*OR* (95%*CI*)
Open wounds of ocular adnexa	115 (10.9)	13 (2.9)	102 (16.9)	0.30 (0.11–0.83)
Superficial wound of eye and adnexa	193 (18.3)	32 (7.1)	161 (26.7)	0.47 (0.19–1.17)
Foreign body on external eye	397 (37.6)	269 (59.4)	128 (21.3)	4.99 (2.13–11.71)
Contusion of eye and adnexa	226 (21.4)	74 (16.3)	152 (25.2)	1.16 (0.48–2.76)
Open wounds of globe	27 (2.6)	8 (1.8)	19 (3.2)	1
Burn confined to eye and adnexa	79 (7.5)	55 (12.1)	24 (4.0)	5.44 (2.09–14.15)
Others	18 (1.7)	2 (0.4)	16 (2.7)	0.30 (0.56–1.60)

*OR* = odds ratio; *CI* = confidence interval

Foreign body on external eye was the most common type for occupational eye trauma, with 269 (59.4%) cases, followed by 74 (16.3%) cases of contusion of eye and adnexa and 55 (12.1%) cases of burn confined to eye and adnexa. However, for patients with non-occupational exposure, there were more superficial wounds of eye and adnexa (161, 26.7%) and contusions of eye and adnexa (152, 25.2%). Patients with occupational exposure were more likely to suffer from foreign body on external eye and burn confined to eye and adnexa. There was no statistically significant difference in the percentage of open wounds of globe between the two groups (*p* > 0.05). The chi-squared test data in Table 3 shows statistically significant correlations between diagnosis of injury and occupational exposure. In the 36–45 age group of occupational eye injury, the most frequent injuries were foreign body on external eye (70.2%), burn confined to eye and adnexa (10.7%), superficial wound of eye and adnexa (6.7%), and contusion of eye and adnexa (6.2%). 

**Figure 5:**
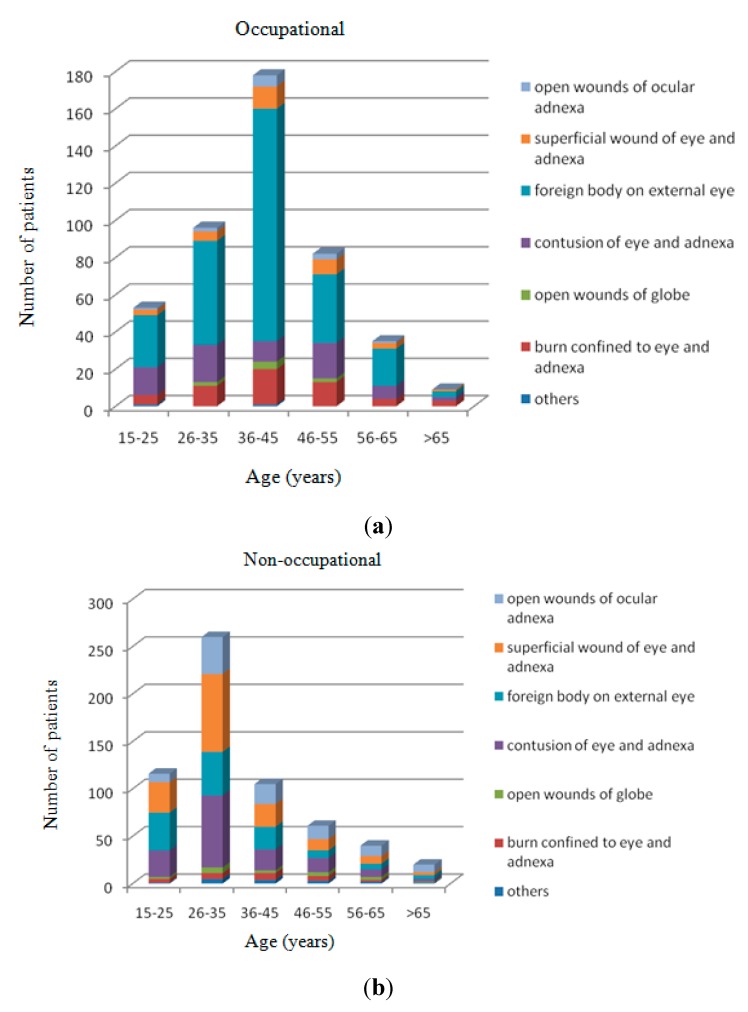
(**a, b**) Frequency of diagnosis of occupational and non-occupational eye injuries by age.

However, the majority of non-occupational eye injuries were within the 26–35 age group (43.2%), for which the most frequent injuries were superficial wound of eye and adnexa (31.5%), contusion of eye and adnexa (29.2%), foreign body on external eye (17.7%), and open wounds of ocular adnexa (15.0%) (Figure 5).

## 4. Discussion

Occupational ocular trauma not only results in economic losses for industry but also affects a considerable number of workers and their families [11]. Despite great progress in therapeutic approaches in recent years, primary prevention remains critically important [12,13,14,15,16]. Our study is the first report on occupational ocular trauma in the Southwest region of China. The data are important for developing effective strategies and programs for work-related eye injury prevention.

The percentages of work-related ocular trauma were reported to range from 38.9% to 73.7% in previous hospital-based epidemiological studies, and the proportion was higher in developing countries [6,11,15,17]. In our study, the percentage of work-related eye injuries (42.9%) is comparable with previously published reports. The mean age of patients suffering from occupational ocular trauma was older than that of non-occupational ocular trauma. The majority of patients with occupational ocular trauma were in the age group of 36 to 45. This could be attributed to the aging of labor in China. The experience of elder workers did not help reduce their exposure to accidents. Consistent with previous clinical studies [18,19,20], our data revealed that eye injuries were far more common in males (occupational 92.1% and non-occupational 71.6%). 

Unlike other regions such as Hong Kong [5], our study showed that most injured workers were in manufacturing (60.7%), followed by construction (19.6%), agriculture (8.2%), and services (4.0%). Because of the limited time and incomplete data collection in the Ophthalmology Department, the description of occupation distribution could be inadequate. However, the trend was apparent that workers in the manufacturing industry had the highest risk of incurring eye injuries. This is not surprising because manufacturing is one of the main industries in this region. Therefore, we suggest that the priorities of health and safety programs should be given to the manufacturing industry.

We also found that temporary workers are more likely to have occupational eye injuries, in agreement with previous studies [11,21]. The reason for this was unclear, but it may relate to the fact that temporary workers usually have a low safety consciousness, low education level, poor work quality, and less training [22]. This makes accidents for temporary workers more frequent than for permanent employees [21]. In order to reduce the number of accidents, health authorities and employers should pay more attention to the management of temporary workers.

Many work-related eye injuries took place in the summer months, July and August. Possible reasons include (1) Chongqing is known as one of China’s “four ovens”, cities where summertime temperatures can soar to 40 °C. Heat and fidgeting increase the incidence of eye injuries. (2) Many factories had their existing workforce put in more hours in summer, when workers feel tired easily. (3) Alcohol consumption is popular in the hot summer months, thus, risks for work-related injury increased. The exact reasons for the peak need to be explored in future studies. There was also a small peak in March and April, the typical hiring time in China, when most of new workers entering the workforce. Meanwhile, the lowest number of work-related eye injuries occurred during the winter months, January and February, the time almost all the factories shut down and workers go back home for the spring festival break. The present study also indicated an incidence peak of occupational eye injuries between 16:00 and 18:00 over a one day period. This is the time right before leaving work. This suggests that the peak may be attributed to a “rushing” phenomenon [17]. Other explanations may be fatigue near the end of the workday, the workflow at job sites, more hazardous work undertaken during this time period. In contrast, we found most non-occupational eye injuries occurred between 11:00 and 13:00, about the middle of the day. Previous studies had shown similar results. More research is needed to better understand the significance of these time-related circumstances with respect to eye injuries. Workers could benefit from better training to recognize fatigue and develop strategies to prevent fatigue from impacting their work, especially while completing hazardous tasks in the late afternoon.

As shown in Table 3, injury from foreign body on external eye and burn confined to eye and adnexa are the two most common occupational eye injuries. On the other hand, superficial wound of eye and adnexa, contusion of eye and adnexa and open wounds of ocular adnexa occurred more frequently in non-occupational eye injuries. This difference in the pattern of injuries matches previously reported studies from other countries [5,11,20]. As is known to all, by wearing protective glasses during work, many types of accidents that cause eye injuries could be easily avoided. Metals, chemicals, electric arcs, hot liquids and glass constitute the majority of the causes in the occupational exposure group (Table 3). As shown in Table 2, most of the occupational exposure patients were temporary workers (65.8%), and they did not wear protective glasses (92.9%) or receive any safety training (77.5%). Age and gender were found to correlate with the susceptibility to ocular trauma. As shown in Figure 5, male workers in the 36–45 age group with injury of foreign body on external eye were the most prominent characteristic in the present study. These workers should be especially targeted to receive eye safety training programs.

An occupational injury involves workers, machines, and materials. There is always a sequence of events or factors that may produce an injury, and any alteration in this sequence or elimination of one of the factors in the injury chain will usually prevent an accident [5]. Safe working conditions and procedures are of vital importance. Regulations on mandatory maintenance and repair of machines and equipment could control the hazard at the source. This need to be implemented by both employers and employees to reduce or eliminate the sources of exposures, and employers should bear the responsibility for providing safe work conditions. Machine guarding is so important that personal protective equipment is sometimes the second line of protection.

Thompson indicated that only 13% of workers were documented as wearing eye protection at the time of injury [23]. A study in Taiwan indicated that eye protection devices could reduce the risk of work-related eye injury by up to 60%, but only 18.4% of workers were wearing eye protection devices when injured [24]. As reported by others, a small percentage of work-related ocular trauma (about 7.1%) occurs while protective eyewear is being worn. This is also supported by our findings. In other words, a large proportion (92.9%) of patients did not wear any protective eyewear, even though protection equipment was available or they believed that such protective devices were required [4,23]. In some circumstances, repeated eye injuries to workers seems the norm rather than the exception [23]. Workers’ attitude toward eye injuries and eye protection is crucial in preventing work-related eye injuries. It is particularly important to implement safety training before work and wear an eye protection during work. This is considered to be an effective way to reduce work-related eye trauma. There also should be compulsory regulations for workers. We believe, with the compulsory regulations in place, work-related ocular trauma can be reduced significantly.

In some developed countries such as the United States, it is mandated that eye injuries be reported to the health care and surveillance system. A Work-Related Injury Statistics Query System was established in these countries [4]. However, many developing countries, including China, do not require employers or health care providers to report preventable injuries. Government staff and public health researchers are thus hindered in their efforts to design effective strategies for trauma prevention due to a lack of epidemiologic data. Therefore, an ocular trauma databank, especially a broad-based trauma databank for further epidemiologic studies would be very useful.

Some limitations exist in our studies. First, like most others on work-related ocular trauma, our investigation was a hospital-based epidemiological research project that does not include data on injuries and illnesses treated outside of the hospital setting. Our findings may not be representative of injuries treated in other medical facilities or injuries where no care was sought at all. Although it is the major eye trauma centre of Beibei District, the Ninth People’s Hospital of Chongqing is not the only designated hospital of the area. As a result, complete data collection was somewhat difficult and estimation of work-related eye injuries was actually limited. Second, eye injury statistics for individuals less than 15 years old were not included in our study, which might have some influence on the findings. In addition, because of the limited research time, information about treatment, follow up, medical costs and the like were not reported in the present study. Such information will be supplemented and provided in future research. However, these limitations do not significantly affect the major findings.

## 5. Conclusions

This study provides insight into the epidemiological characteristics of occupational ocular trauma in the Southwest region of China. Our research indicated that male worker, low education level, lack of safety training, without machine guarding and eye protection were significant risk factors for work-related eye injuries. The current findings might be considered as a baseline for future research on regional work-related eye injuries. Our findings will provide valuable information for further development of preventive strategies.
